# Panoptic quality should be avoided as a metric for assessing cell nuclei segmentation and classification in digital pathology

**DOI:** 10.1038/s41598-023-35605-7

**Published:** 2023-05-27

**Authors:** Adrien Foucart, Olivier Debeir, Christine Decaestecker

**Affiliations:** 1 Laboratory of Image Synthesis and Analysis, École polytechnique de Bruxelles, Université Libre de Bruxelles (ULB), 1050 Brussels, Belgium; 2 Center for Microscopy and Molecular Imaging (CMMI), Université Libre de Bruxelles (ULB), Gosselies, Belgium

**Keywords:** Biomedical engineering, Image processing

## Abstract

Panoptic Quality (PQ), designed for the task of “Panoptic Segmentation” (PS), has been used in several digital pathology challenges and publications on cell nucleus instance segmentation and classification (ISC) since its introduction in 2019. Its purpose is to encompass the detection and the segmentation aspects of the task in a single measure, so that algorithms can be ranked according to their overall performance. A careful analysis of the properties of the metric, its application to ISC and the characteristics of nucleus ISC datasets, shows that is not suitable for this purpose and should be avoided. Through a theoretical analysis we demonstrate that PS and ISC, despite their similarities, have some fundamental differences that make PQ unsuitable. We also show that the use of the Intersection over Union as a matching rule and as a segmentation quality measure within PQ is not adapted for such small objects as nuclei. We illustrate these findings with examples taken from the NuCLS and MoNuSAC datasets. The code for replicating our results is available on GitHub (https://github.com/adfoucart/panoptic-quality-suppl).

## Introduction

The notion of “Panoptic Segmentation” (PS) and its corresponding evaluation metric, “Panoptic Quality” (PQ), were introduced by Kirillov et al. in 2019^1^. Panoptic segmentation, per Kirillov’s definition, attempts to unify the concepts of semantic segmentation and instance segmentation into a single task with a single evaluation metric. In PS tasks, some classes are considered as stuff (meaning that they are regions of similar semantic value, but with no distinct instance identity, such as “sky” or “grass”), and some as things (i.e., countable objects). The concept was initially applied to natural scenes using the Cityscapes, ADE20k and Mapillary Vistas datasets. It was then applied to the digital pathology task of nuclei instance segmentation and classification in Graham et al’s 2019 paper that introduced the HoVer-Net deep learning architecture^2^.

PQ was then adopted as the ranked metric of the MoNuSAC 2020 challenge^3^, then the CoNIC 2022 challenge^4^, and was used in several recent publications as a means of comparison with the state-of-the-art^5–9^ and/or to make choices in algorithm development^10^. Yet, as we show in our analysis of the MoNuSAC results^11^, this metric can hide much useful information about the performance of competing algorithms. In this work, we analyse more generally why the PQ metric is not suitable for cell nucleus instance segmentation and classification and should therefore be avoided. In particular, we demonstrate the following:

- PQ is used in digital pathology on *instance segmentation and classification* tasks, but these tasks are fundamentally different from the *panoptic segmentation* task for which the metric was designed.
- The reliance on the *Intersection over Union* segmentation metric, both as a *matching rule* and as a contributor to PQ computation, is not appropriate for segmentation of cell nuclei, due to the small size of these objects.
- Summarising the performance of a complex, multi-faceted task into a single, entangled metric leads to *poorly interpretable results*.

We first use a theoretical approach to explain the aforementioned problems. We then use examples from public challenges and benchmark datasets to demonstrate their effect.

## Definitions

In the definition of a “Panoptic Segmentation” problem by Kirillov et al.^1^, each pixel in an image can be associated with both a ground truth class, *c*, and a ground truth instance label, *z*. A pixel cannot have more than one class or instance label (i.e. no label overlap is allowed), but a pixel does not necessarily have an instance label (i.e. *z* can be undefined). The distinction between *things* and *stuff* is that *stuff* are classes that do not require instance labels, while *things* are classes that do.

PQ considers each class separately. For each class *c*, $G_c = \{g_k\}$ is the set of ground truth instances of the class (for *stuff*, there is only one element in the set since there are no distinct instances). Given the set of predictions for this class, $P_c = \{p_l\}$, $PQ_c$ is computed as follows.

First, the matches between the ground truth instances and the predicted instances are determined for class *c*. A match is defined as a pair $(g_k, p_l)$ such that the Intersection over Union verifies $IoU(g_k, p_l) = \frac{|g_k \cap p_l|}{|g_k \cup p_l|} > 0.5$, where |.| is the cardinality of the set.

Second, using this strict matching rule, each segmented instance in $G_c$ and $P_c$ is assigned to one of the following three sets: True Positives (TP), False Positives (FP) and False Negatives (FN), which are defined as follows:

$$\begin{aligned} TP= & {} \{(g_k, p_l); IoU(g_k, p_l) > 0.5\}\\ FP= & {} \{p_l; IoU(g_k, p_l) \le 0.5 \forall g_k\}\\ FN= & {} \{g_k; IoU(g_k, p_l) \le 0.5 \forall p_l\} \end{aligned}$$

The strict matching rule ensures that for a given ground truth object instance, $g_k$, there can only be one corresponding predicted instance, $p_l$.

Finally, *PQ* for class *c* in image *i* is computed as:

$$\begin{aligned} PQ_{c,i} = \frac{\sum _{(g_k, p_l) \in TP} IoU(g_k, p_l)}{|TP| + \frac{1}{2} |FP| + \frac{1}{2} |FN|} \end{aligned}$$

which can be decomposed into:

$$\begin{aligned} RQ_{c,i}= & {} \frac{|TP|}{|TP|+\frac{1}{2} |FP| + \frac{1}{2} |FN|}\\ SQ_{c,i}= & {} \frac{\sum _{(g_k, p_l) \in TP} IoU(g_k, p_l)}{|TP|}\\ PQ_{c,i}= & {} SQ_{c,i} \times RQ_{c,i} \end{aligned}$$

$RQ_{c,i}$, the “Recognition Quality” of Kirillov et al.^1^, corresponds to the per-object $F_1$-score of class *c* in image *i*, and $SQ_{c,i}$, the “Segmentation Quality”, is the average IoU computed on the matched pairs of ground truth and predicted instances of this class. In digital pathology, *RQ* is also often referred to as the Detection Quality, and therefore noted as *DQ* by Graham et al.^2^. As explained in the next section, these same definitions of *RQ* and *DQ* have different impacts depending on the precise nature of the task.

Different choices were made to aggregate the per-image, per-class $PQ_{c,i}$ into a single “multi-class average *PQ*”. In the original HoVer-Net publication^2^, the MoNuSAC challenge^3^ and the Lizard dataset publication^6^, a multi-class $PQ_i$ is computed for each image as $PQ_i = \frac{1}{m_i} \sum _{c=1}^{m_i} PQ_{c,i}$, where $m_i$ is the number of classes present in image *i*. The average *PQ* is then computed on the *n* images as:

$$\begin{aligned} aPQ = \frac{1}{n}\sum _{i=1}^n PQ_i \end{aligned}$$

In contrast, in the more recent CoNIC challenge^4^, *TP*, *FP*, *FN* and *IoU* are computed for each class over the images in the dataset, so that $PQ_c$ is computed over all images merged together, and the final average *PQ* is simply:

$$\begin{aligned} mPQ = \frac{1}{m}\sum _{c=1}^m PQ_c \end{aligned}$$

**Figure 1:**
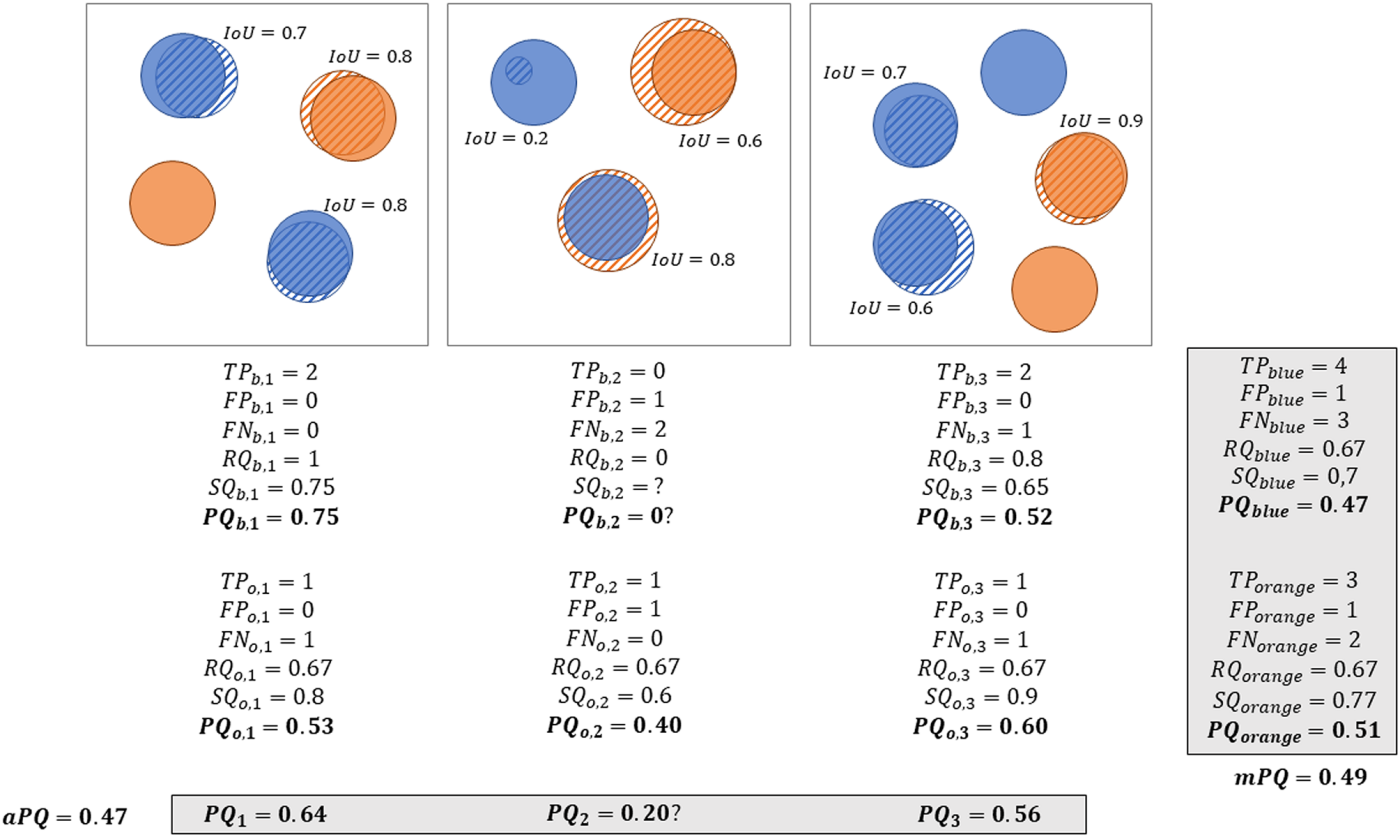
Illustration of the process of computing PQ on a set of 3 images, with the two different aggregation methods: bottom, *aPQ* is computed for each image based on the per-class values; right, the individual components (TP, FP, FN, IoUs) are aggregated on all images before computing *mPQ*. The ground truth and predicted masks are represented by solid and hatched discs, respectively. The b and o indices indicate the blue and orange classes, respectively. It should be noted that in the second image, the prediction with an IoU of 0.2 is unmatched (based on the 0.5 threshold) and thus counts as one blue FP and one blue FN. The misclassified object counts as one blue FN and one orange FP.

These processes are illustrated in Fig. 1. This figure also illustrates one of the potential issues of the first aggregation method above, namely dealing with missing classes in the annotations or in the predicted objects for an image. In the second image in Fig. 1, there are no correctly predicted blue objects, which means that for this class, there are no True Positives, and thus SQ is undefined. It seems logical that if there is either a ground truth object and no prediction, or a prediction and no ground truth, the resulting PQ should be 0. However, this is not a result that follows directly from the definition.

## Theoretical analysis

### Panoptic segmentation vs instance segmentation and classification

The first problem with using *PQ* for assessing nuclei instance segmentation and classification is that it is not a panoptic segmentation task. Panoptic segmentation is characterized by two key factors:

- Every pixel is associated with one single *class label*.
- Every pixel is associated with one single optional *instance label*.

**Figure 2:**
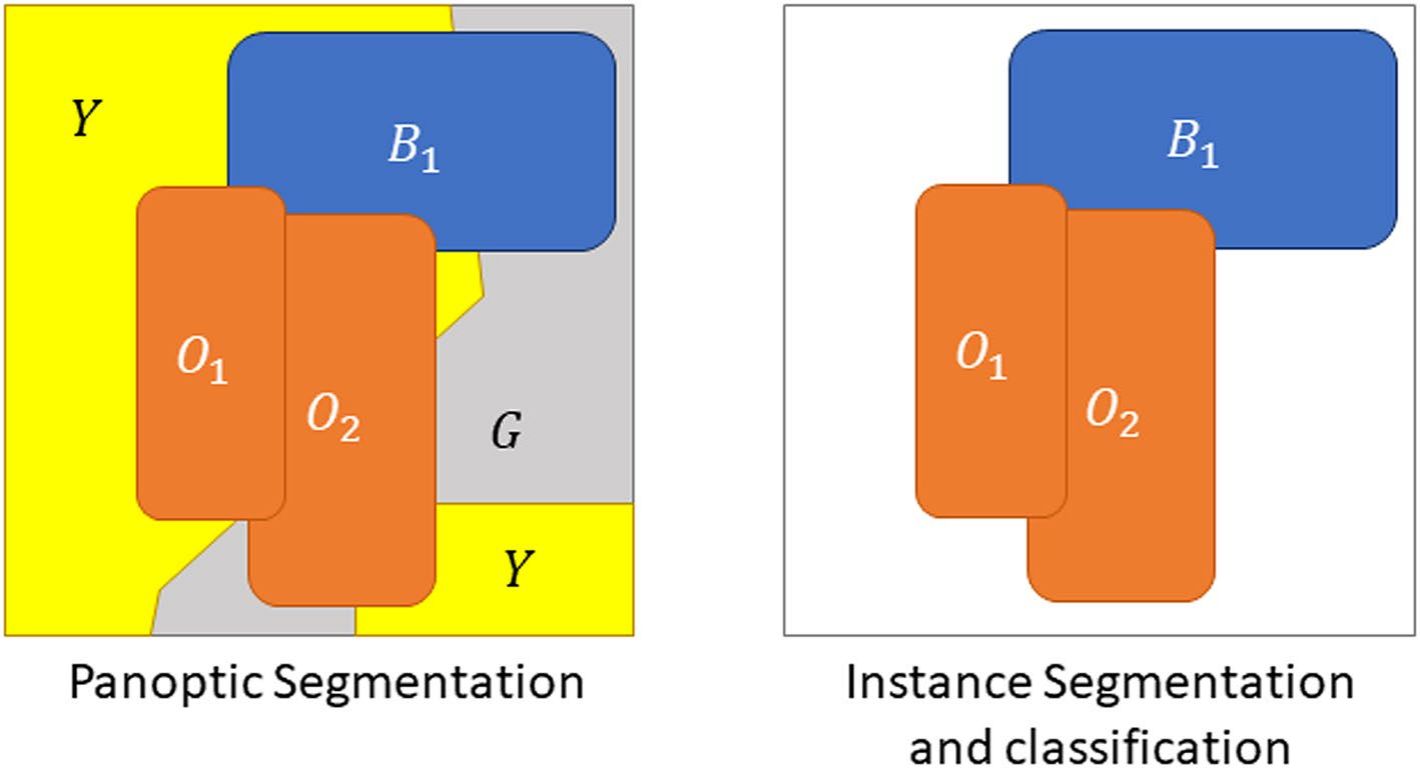
Difference between a PS and an ISC task. In the former, every pixel of the image is associated to a class and an optional instance. Some classes (“stuff”) always count as a single instance, even if disjointed (Y and G on the left). In an ISC task, however, it is possible for pixels to have neither class nor instance and be part of the “background” (in white).

In ISC, the class label is *also optional*, as there is usually a “background class” that corresponds to everything that is not an object of interest. For nucleus ISC, this class may include tissue-free regions, but in a dataset like MoNuSAC it mainly includes tissue regions without objects of interest (i.e., cytoplasmic and extracellular regions, as well as nuclei that are not from one of the target classes.). Additionally, if a pixel is associated with a class label, it also needs to have an instance label (i.e. there is no *stuff*, only *things*, using Kirillov’s terminology), as illustrated in Fig. 2.

This is not necessarily a problem in itself. Metrics can find uses outside their original, intended scope: IoU is generally traced to Paul Jaccard’s study of flora distribution in the Alps^12^, long before “image segmentation” was on anyone’s radar. However, the transition between PS and ISC tasks is problematic in this case. The “Recognition Quality” in Kirillov’s definition corresponds to the *classification* F1-score, whereas the “Detection Quality” in Graham’s definition is the *detection* F1-score. The definitions appear identical, but there is actually a key difference. In the classification F1-score, everything is assumed to have a class. The object-level confusion matrix therefore looks like this (here for 3 classes, with in row the ground truth classes and in column the predictions):

$$\begin{aligned} \begin{pmatrix} CM_{11} &{}\quad CM_{12} &{}\quad CM_{13}\\ CM_{21} &{}\quad CM_{22} &{}\quad CM_{23}\\ CM_{31} &{}\quad CM_{32} &{}\quad CM_{33} \end{pmatrix} \end{aligned}$$

In this case, if the predictions provided by different algorithms on the same dataset are compared, the sum of the elements of each of the corresponding matrices $S = \sum _{ij} CM_{ij}$ remains constant, which is not the case for detection as explained below.

In a *detection* F1-score, an additional “background” class has to be considered. It is therefore possible that predicted objects do not to belong to any target class and that ground truth objects have no matched prediction. The confusion matrix for a 3 classes problem is therefore a 4x4 matrix (in row the ground truth classes and in column the predictions):

$$\begin{aligned} \begin{pmatrix} N.C. &{}\quad CM_{01} &{}\quad CM_{02} &{}\quad CM_{03}\\ CM_{10} &{}\quad CM_{11} &{}\quad CM_{12} &{}\quad CM_{13}\\ CM_{20} &{}\quad CM_{21} &{}\quad CM_{22} &{}\quad CM_{23}\\ CM_{30} &{}\quad CM_{31} &{}\quad CM_{32} &{}\quad CM_{33} \end{pmatrix} \end{aligned}$$

Class 0 being background, the top-left element is “Not Countable” because there are no countable and correctly predicted “background objects”. The first row corresponds to false positive detections (predicted objects with no corresponding ground truth) and the first column to false negative detections (target objects completely missed). In this case, the sum of the elements of the matrix is no longer constant between different algorithms tested on the same dataset because the sum of the first row depends only on the (false) detections of each algorithm. In contrast, the sum of each of the subsequent rows is determined by the ground truth class distribution in the dataset and thus remains constant between the algorithms.

This may seem like a relatively minor issue, but it adds a lot of confusion to the interpretability of the PQ metric. The original PQ mixes classification and segmentation, but both can be separately analysed in the RQ and SQ. However, PQ applied to ISC mixes classification and detection within RQ, making it even more difficult to understand why one algorithm scores better than another.

What may be more problematic is that PQ is computed per class and, as illustrated in Fig. 1, assigns a greater penalty to a *good detection* with a *wrong class* (which is counted as a “false negative” for the ground truth class, and as a “false positive” for the predicted class) than to a *missed detection* (which is only a “false negative” for the ground truth class).

### Intersection over Union for digital pathology objects

The IoU metric does not seem a priori controversial for evaluating a segmentation task. It is widely used, including in many digital pathology challenges and benchmarks^13^. However, it also has known weaknesses, particularly when used on small objects^14^.

As previously defined, the IoU between ground truth object $g_k$ and predicted object $p_l$ can be expressed as:

$$\begin{aligned} IoU(g_k, p_l) = \frac{|g_k \cap p_l|}{|g_k \cup p_l} \end{aligned}$$

Another way to compute it is to first define the per-pixel TP, FP and FN as:

$$\begin{aligned} TP(g_k, p_l)= & {} |g_k \cap p_l|\\ FP(g_k, p_l)= & {} |\lnot g_k \cap p_l|\\ FN(g_k, p_l)= & {} |g_k \cap \lnot p_l| \end{aligned}$$

where $\lnot$ denotes the elements that are outside of a set of pixels. IoU can then be written as:

$$\begin{aligned} IoU(g_k, p_l) = \frac{TP(g_k, p_l)}{TP(g_k, p_l) + FP(g_k, p_l) + FN(g_k, p_l)} \end{aligned}$$

The problem with IoU comes from the combination of two different characteristics which are very common in digital pathology objects:

- The *exact borders* of the object are very often fuzzy and ill-defined.
- The *area* (i.e. number of pixels) of the object can be very small, as in the case of cell nuclei.

Because of (a), any predicted segmentation, even accurate, is likely to have some misalignment around the boundary of the object. This tends to make FP and FN correlated to the perimeter of the object while TP tends to be correlated with the object area. As the $\frac{Perimeter}{Area}$ ratio is generally higher for small objects, the corresponding IoU value therefore tends to be lower, even for a very good segmentation. For objects that are very small even at high levels of magnification, such as nuclei, this can lead to very problematic results, as we show in our experiments below.

This problem is compounded by the fact that IoU does not weight overestimation and underestimation of the object size in the same way. If we imagine a perfectly matching prediction, and then add *n* pixels from outside of the object to the predicted set, the corresponding “overestimated IoU” is $IoU^+ = \frac{TP}{TP+n}$. If, however, we *remove* *n* pixels from the set of true positives, we end up with $IoU^- = \frac{TP-n}{TP+n}$, as these removed pixels will count both as “less true positives” and “more false negatives”. In the “overestimated” case, they would count as “less true negatives”, but those have no impact on IoU. For an object with an area of 150 px, for instance, an overestimation of 50 px of its size would lead to an IoU value of 0.75, whereas an underestimation of 50 px would lead to an IoU value of 0.5.

These IoU properties impact PQ at two different levels: the *matching rule* and *segmentation quality*. Regarding the matching rule, the conjunction of a small object and an algorithm that underestimates its size can easily lead to erroneous “false detections”, where clearly matching objects are rejected due to an IoU value under 0.5. Regarding segmentation quality, the problem lies with interpretability and class averaging. When object sizes vary between classes, the limits of what would constitute a “good” IoU value should differ between classes. Calculating an average PQ across classes (see Fig. 1) therefore adds hidden “weights” to the metric. Indeed, algorithms that perform poorly on classes with smaller objects necessarily tend to have a lower average IoU (and therefore PQ) value than those that perform poorly on classes with larger objects.

Additionally, it is well known that IoU does not consider the shape of the object (like other overlap-based metrics such as the Dice Similarity Coefficient). As demonstrated by Reinke et al.^14^, predictions that miss the shape of the object completely can end up with the same IoU value as those that match the shape well, but are slightly offset, or slightly under- or overestimate the object size. To give a clearer meaning to the segmentation performance of an algorithm, it is often useful to refer to both an overlap-based metric such as IoU and a border distance metric such as Hausdorff’s Distance (HD). Thus, using PQ completely misses an important aspect of assessment. In digital pathology tasks, the shape of the object of interest is often very relevant to clinical and research applications that rely on image analysis. It is therefore ill-advised to base the choice of an algorithm on a metric that ignores this particular aspect.

### Interpretability of the results

As we have shown in a previous work^11^, the PQ metric hides a lot of potentially insightful information about the performances of algorithms by merging information of a very different nature. While SQ and RQ have the same range of possible values, being bounded between 0 and 1, multiplying these values to get PQ implies that the impact of a change in SQ by a factor *a* is exactly the same as a change of RQ by the same factor.

The significance of these changes for the underlying clinical applications, however, can be very different. As shown above, a 10% reduction in SQ may only indicate a small size underestimation for each segmented object (which for small objects would probably be within the typical interobserver variability range), whereas a 10% reduction in RQ indicates potentially much more significant errors, with entire objects being added as false positives, or missed as false negatives. The interpretation of the relative change in SQ is dependent on the size of the ground truth objects, while the interpretation of the relative change in RQ is more likely to depend on the class distribution. Ranking different algorithms with PQ therefore leads to results that cannot really be related to clinical application needs.

## Experimental analysis

### Material and methods

To show the concrete impact of our theoretical analysis, we select two public digital pathology datasets designed for instance segmentation and classification: NuCLS^15^, and the MoNuSAC challenge dataset^3^.

#### NuCLS dataset and experiments

The NuCLS dataset^15^ proposes a “crowdsourced” dataset where the annotations are made by non-pathologists from algorithmic suggestions, and with corrections by junior and senior pathologists. It also provides a “multi-rater” dataset, where detailed individual annotations from experts and non-experts are provided on selected FOVs. The objects of interest are nuclei in breast cancer tissue, and all images and annotations are provided with a resolution of around 0.25 microns-per-pixel (40 $\times$ magnification). There are 13 “raw classes”, which are then hierarchically grouped into 7 “classes” and then 4 “super-classes”. All slides were stained with Haematoxylin & Eosin (H&E), and were obtained from the TCGA (The Cancer Genome Atlas) archives.

Using the raw annotations from the evaluation dataset, we select all the pathologists (junior and senior) and extract all their detailed annotations (excluding annotations where only the bounding box is provided). Then, for each pair of experts, we identify all pairs of matched annotations. We define a match here in the loosest possible sense, i.e. as any overlapping pair of annotated objects. If multiple matches are found for a single object, we select the match with the largest IoU. We then look at the relationship between experts’ IoU and object area.

To better visualise the IoU sensitivity to small differences in overlap, we also select a single nucleus and the ground truth given by one of the senior pathologists to compare with the other proposed segmentations, all of which would be considered “correct” from a clinical perspective. We measure the corresponding IoU and HD to examine their relationship. HD is the maximum distance between any point in the contour of an object and its closest point in the contour of the other object.

#### MoNuSAC dataset and experiments

The MoNuSAC challenge dataset^3^ includes annotations for nuclei of four different classes (epithelial, lymphocyte, neutrophil and macrophage) from tissue sampled in different organs (breast, kidney, lung, prostate). All slides were stained with H&E and, like in NuCLS, are sourced from the TCGA archives and are presented at a resolution of around 0.25 mpp. Two different aspects are interesting to explore with the publicly available training and test data. First, there is a large difference in set size and nucleus size between the different classes. Second, the detailed predictions made on the test set by the algorithms of four participating teams are available, which allows us to directly examine how PQ (whatever the aggregation method used) penalises different types of error in a real challenge setting.

We therefore conduct the following experiments on the MoNuSAC test set.

Based on the ground truth annotations, we create three different slightly modified versions of the annotation masks: one with a single-pixel *dilation*, one with a single-pixel *erosion*, and one with a single-pixel *vertical shift* of the whole masks. In all three cases, those modified versions would not be “worse” than the original and fall well within the variability caused by the fuzziness of the contours. We compute IoU of each of those modified objects against that of the original ground truth and look at the relationships between IoU, object area and class.

We then examine selected examples from participants’ predictions to see how their errors were penalised, and where PQ may lead to a ranking that does not really match the performance of the algorithms in terms of “usefulness” for clinical and research practice.

### Results

#### NuCLS experiments

**Figure 3:**
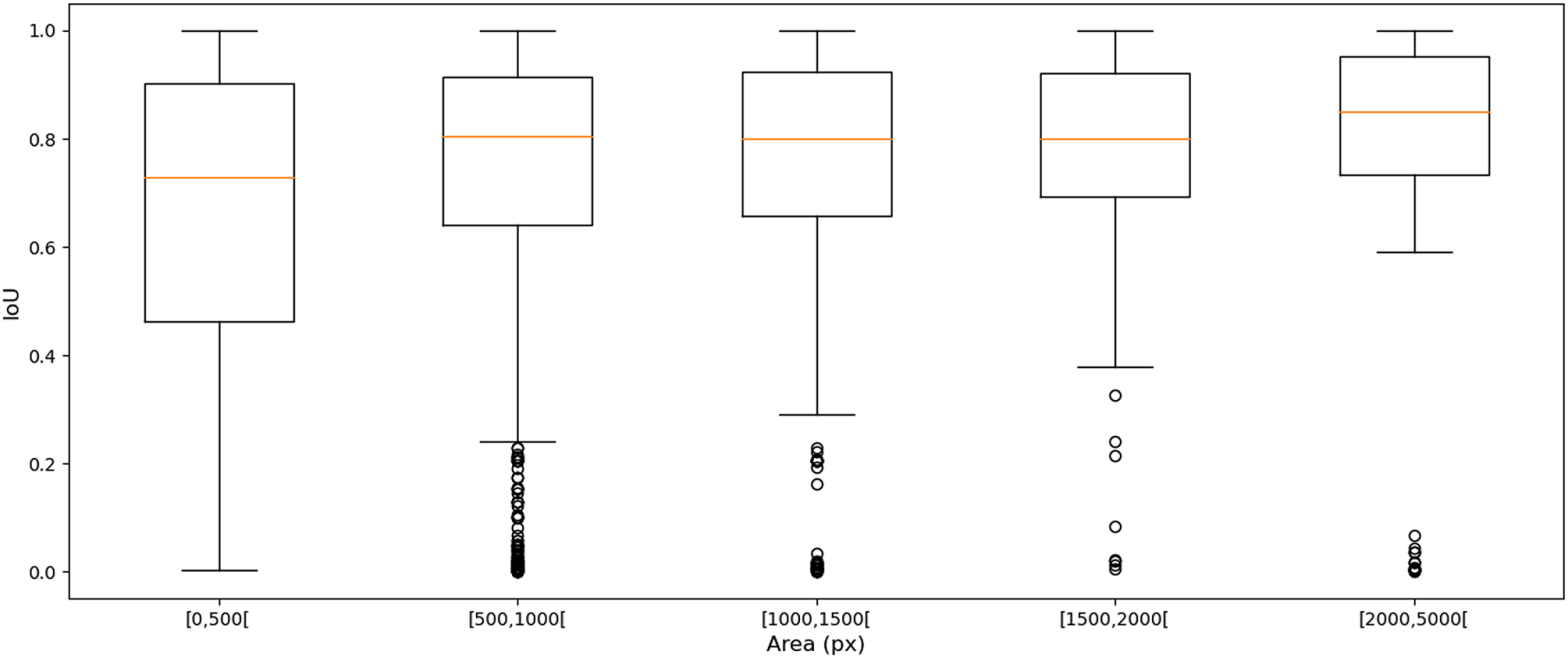
Distribution of the inter-expert IoU values based on the raw “multi-rater” annotations of the NuCLS dataset, in relation with the object sizes. The orange line is the median, and the boxes show the inter-quartile range. The bars show the minimum-maximum range, excluding outliers.

Figure 3 shows the relationship between the distribution of the inter-expert IoU values and the object sizes. For smaller objects, it is much more common to observe smaller IoU values, which do not necessarily correspond to “bad” segmentations but rather disagreements or inaccuracies about the exact location of object boundaries, which is inevitable given the fuzzy nature of nucleus contours.

**Figure 4:**
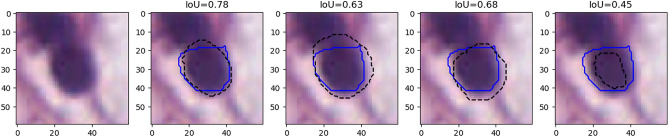
Nucleus from the NuCLS dataset, with examples of four different proposed “good” segmentations (dashed black lines), with their IoU value measured against the ground truth of one of the senior pathologists (solid blue lines).

This result is illustrated on a single cell in Fig. 4. We show four different segmentations compared to the ground truth provided by one of the experts (blue line). All four segmentations are arguably “as good‘” as the ground truth, as the exact contours are impossible to determine due to fuzziness (and compression artefacts). IoU, however, has relatively low values, such as 0.78, 0.63, 0.68 and 0.45, with the latter falling under the 0.5 threshold needed to be considered a “match” by the PQ metric.

**Figure 5:**
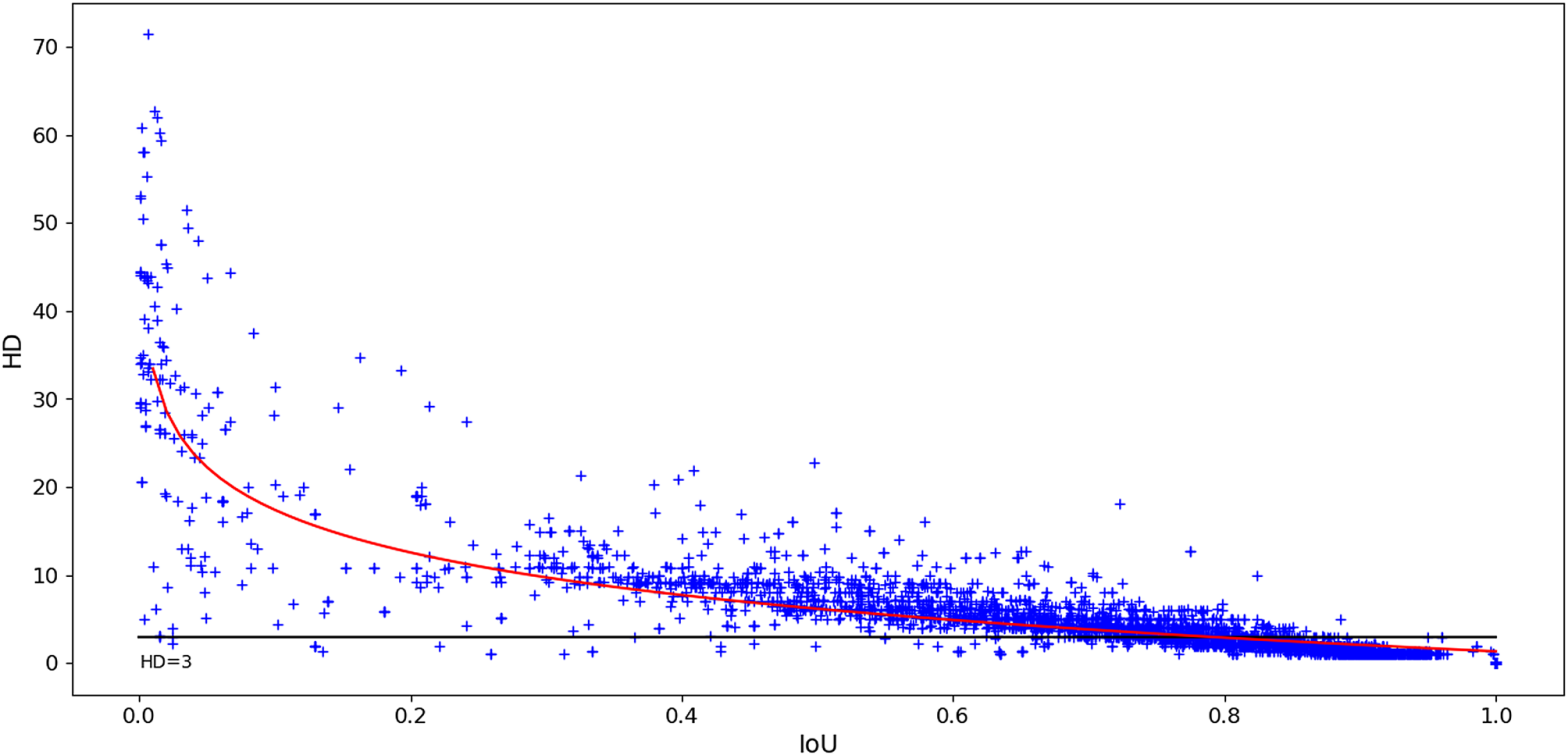
Relationship between IoU and HD for all inter-expert overlapping pairs of objects in the NuCLS multi-rater dataset annotations. The horizontal line indicates a HD value of 3 px. The red line shows the best log-linear regression fit between IoU and HD.

The relationship between IoU and HD on all overlapping pairs of expert annotations is plotted in Fig. 5. While there is a general tendency to observe an inverse relationship between HD and IoU, this is very dispersed for low IoU values and starts to flatten out from an IoU value of 0.3. For instance, the whole region with IoU values between $\sim$ 0.3 and $\sim$ 0.5 corresponds mostly to HD values of about 10 (the outliers with $HD < 3$ and $IoU < 0.5$ correspond to incorrect annotations that contain only a few pixels). Many pairs with IoU values of about 0.7 or larger have HD values less than 3 px (horizontal line), meaning that no point on one contour is more than 3 px (or $\sim$ 0.75 $\upmu$m) away from the other contour. This indicates that many predictions within a wide range of IoU values are actually very similar to the ground truth.

#### MoNuSAC experiments

**Figure 6:**
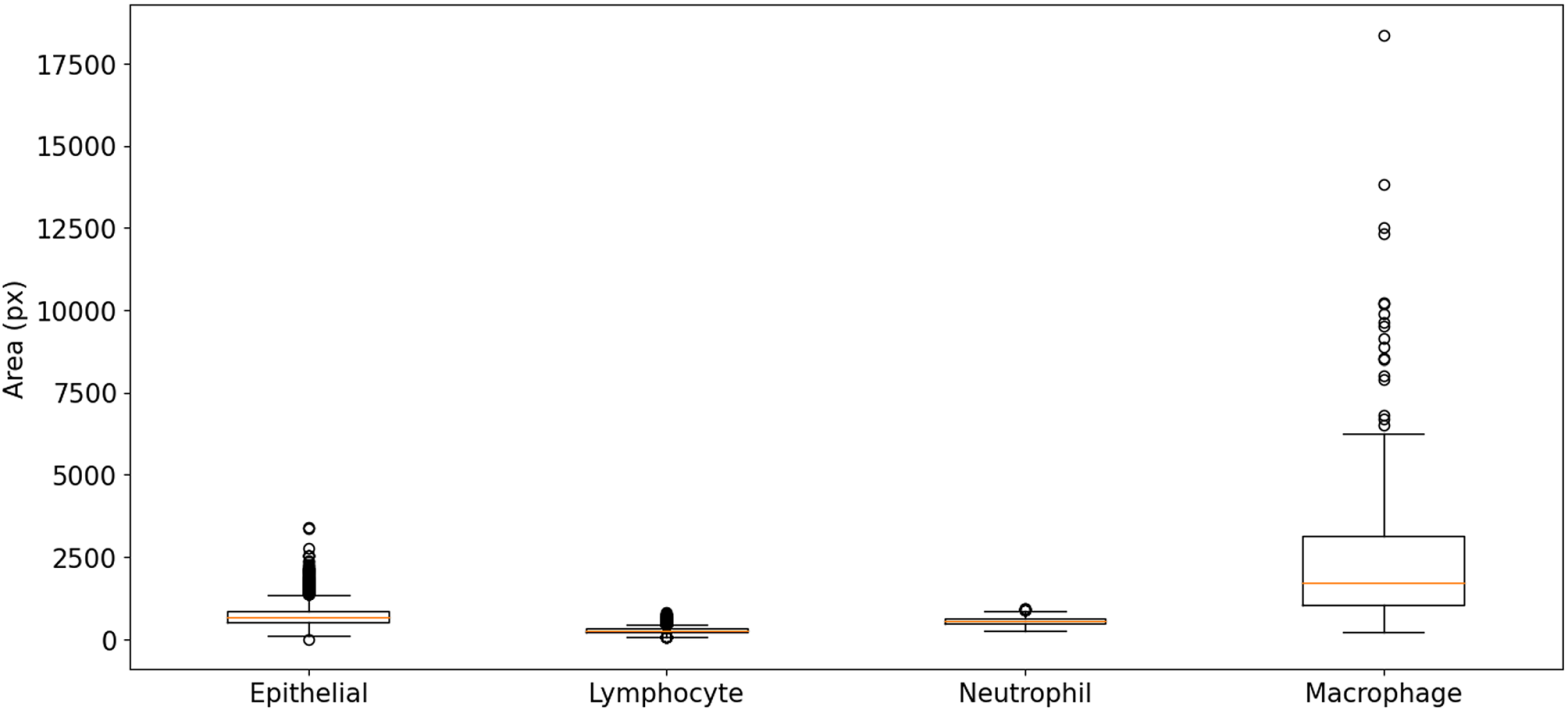
Nucleus area distribution based on the classes of the MoNuSAC test set (for boxplot meaning, see Fig. 3).

The four classes of the MoNuSAC dataset have very different nucleus size distributions, as evidenced in Fig. 6. Lymphocytes are the smallest (median area = 266 px, interquartile range = [221–314 px]), followed by neutrophils (546 px [468–627 px]) and epithelial nuclei (683 px [524–858 px]), with macrophages much larger than the three others and with a very wide distribution (1734 px [1032–3152 px]).

**Figure 7:**
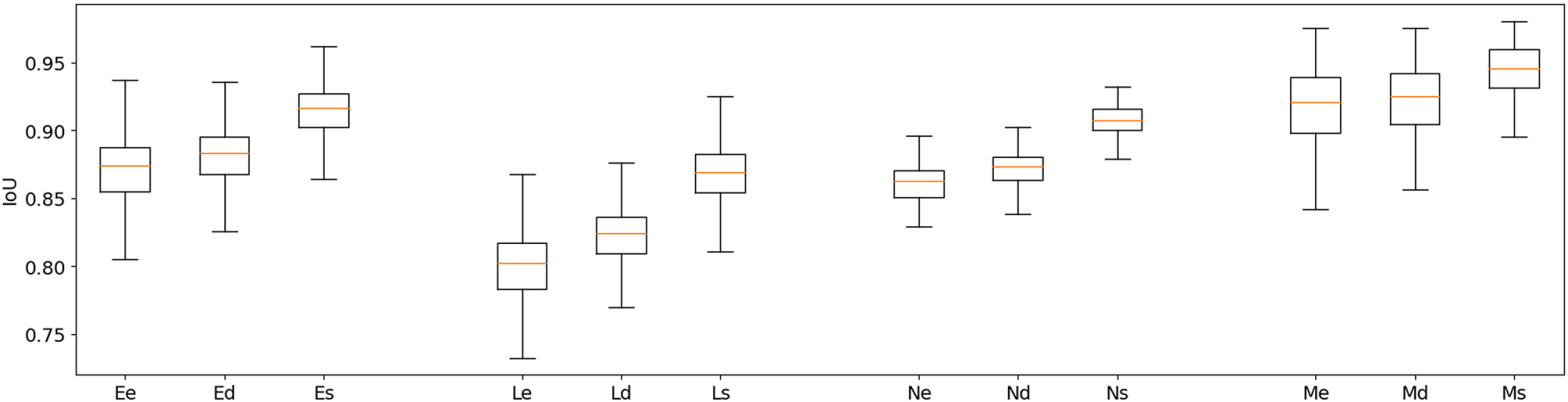
IoU distribution for the Epithelial (E), Lymphocyte (L), Neutrophil (N) and Macrophage (M) classes after a single-pixel erosion (e), dilation (d) and vertical shift (s). Outliers in the epithelial cells with IoU $< 0.5$ are not shown and correspond to mistakes in the annotations with only small parts of the nuclei being contoured (for boxplot meaning, see Fig. 3).

The effect of the single-pixel erosion, dilation, and vertical shift on IoU are shown in Fig. 7. Three findings emerge clearly from these distributions. First, the effect on IoU of small object edge perturbation is clearly stronger for smaller object classes. For the lymphocytes, the single-pixel erosion leads to a median IoU of 0.80, compared to 0.92 for the macrophages. Second, even for the comparatively larger macrophages, the resulting “error” on PQ introduced by the edge uncertainty is still quite large. An IoU value of 0.92 means that the penalty for not perfectly matching the annotator’s exact delineations is the same as completely missing 8% of the objects of this class. Finally, we can see the effect of the bias towards “overestimation”, as the single-pixel dilations have always slightly higher IoU values than the single-pixel erosions, with a more pronounced effect for smaller objects (e.g. for the lymphocytes median IoU of 0.82 for the dilations, compared to 0.80 for the erosions). Since the effect depends on the size of the objects in pixels, it is even more pronounced when working at lower resolutions. Repeating the single-pixel erosion and dilation on the image downscaled by a factor of two (to simulate a 20$\times$ magnification image, as used for instance in the CoNiC challenge^4^ leads to median IoUs of 0.64 (erosion) and 0.71 (dilation) for the lymphocytes, a much larger bias compared to the original results at 40$\times$ magnification.

**Figure 8:**
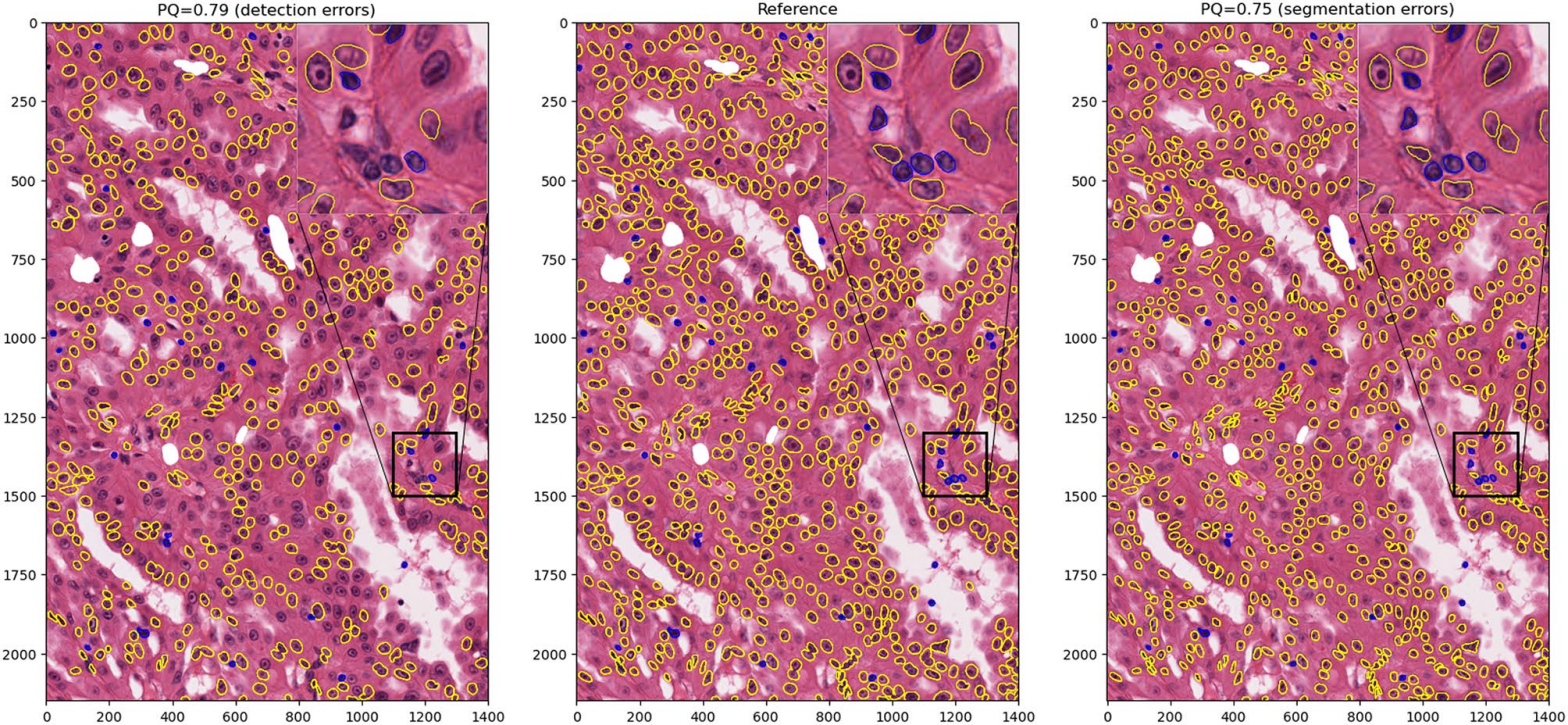
Illustration of the difficulty of interpreting the PQ score, based on an image with 861 annotated nuclei from two classes (epithelial in yellow, lymphocytes in blue). The central image shows the reference annotations from the MoNuSAC challenge. On the left, a synthetic “prediction” with around 40% detection errors. On the right, a synthetic “prediction” with segmentation errors (around 30% removed tissue area, which correspond to an erosion of only 2 or 3 pixels on each nucleus). On top, the corresponding PQ scores. Regions marked as “ambiguous” in the annotations have been whitened out.

Clearly, similar values for RQ and SQ should be interpreted completely differently, even though their contribution to the overall PQ score is the same. We illustrate that on a large image patch from the MoNuSAC challenge (see Fig. 8): a prediction that misses around 40% of the objects in the image (but perfectly segments all others) can have a higher PQ value that a prediction that removes around 30% of each object’s area (but perfectly detects all of them), even though missing objects is clearly much worse than underestimating their size for any potential use in pathology.

**Figure 9:**
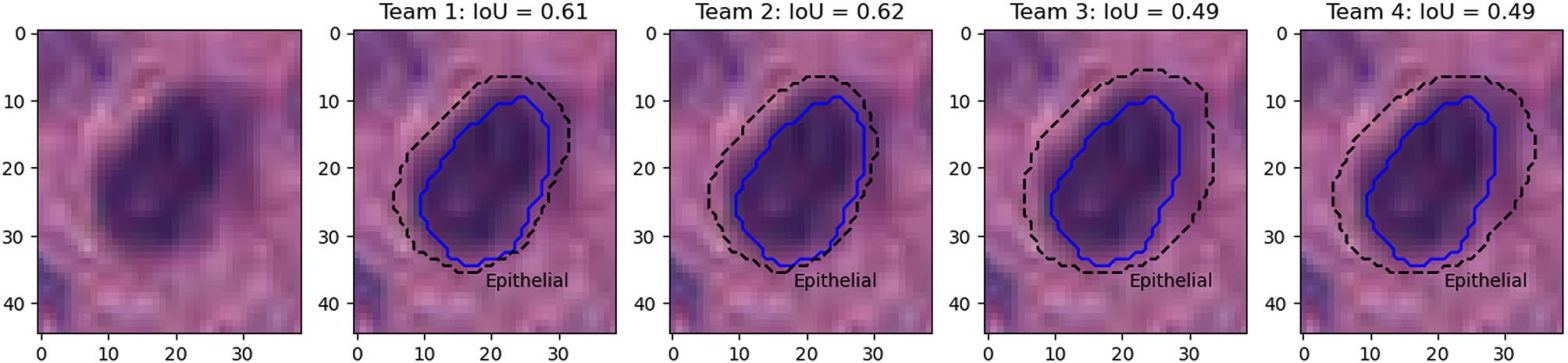
Nucleus of an epithelial cell from the MoNuSAC dataset, with predictions from the four teams (dashed line) and the corresponding IoU values with the ground truth segmentation (solid blue).

**Figure 10:**
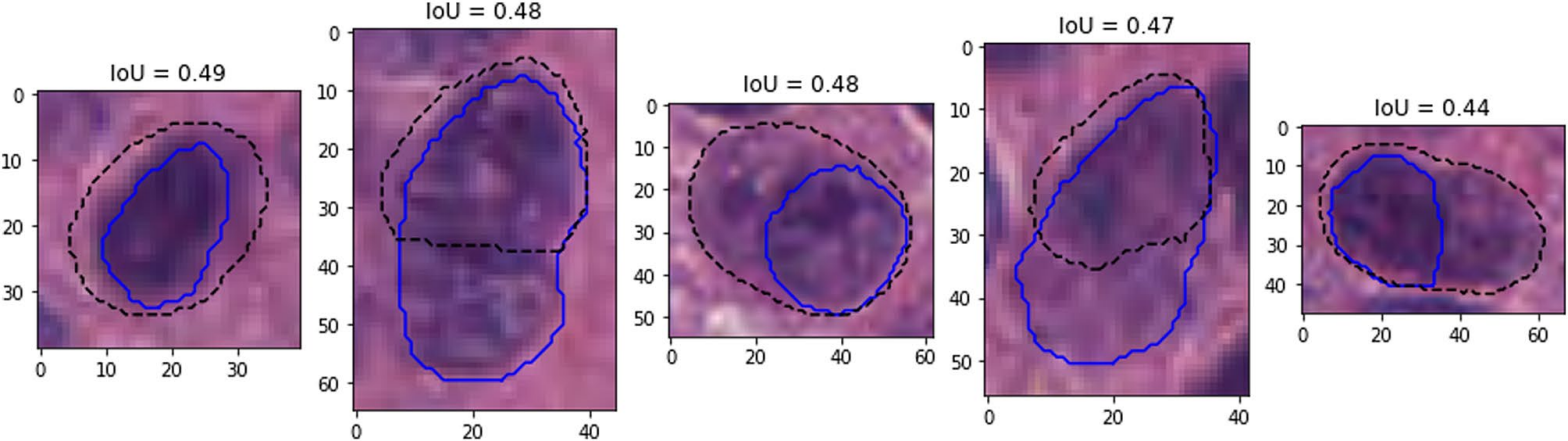
Several predictions from team 4 (dashed black line) with corresponding ground truth annotations (solid blue line) on images from the MoNuSAC test set and not counted as matches due to IoU values less than 0.5.

**Figure 11:**
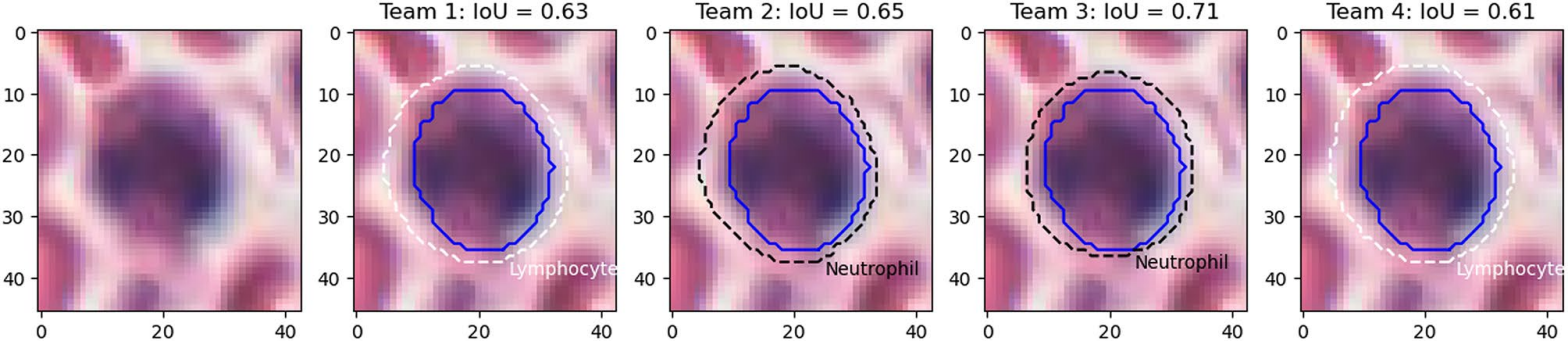
Nucleus of a neutrophil, with predictions from the four teams (dashed line) and the corresponding IoU value computed with the ground truth segmentation (solid blue). Black lines are used for correct classifications, white lines for incorrect classifications.

**Figure 12:**
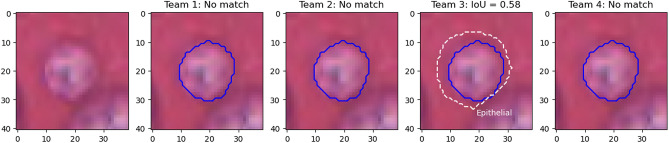
Nucleus of a macrophage, with predictions of the four teams (dashed line) and the corresponding IoU values computed with the ground truth segmentation (solid blue). Black lines are used for correct classifications, white lines for incorrect classifications.

**Figure 13:**
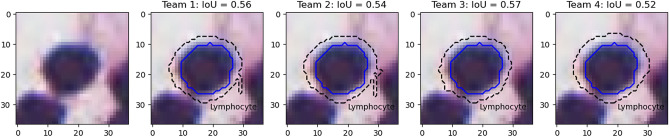
Nucleus of a lymphocyte, with predictions of the four teams (dashed line) and the corresponding IoU values computed with the ground truth segmentation (solid blue).

In addition, we use prediction results from the MoNuSAC challenge to illustrate some of the problems associated with using the PQ metric to evaluate nucleus instance segmentation and classification. Figs. 9, 10, 11, 12 and 13 show the predictions of the four teams whose detailed results are available from the challenge website on selected examples from each class.

Figure 9 shows the results on an epithelial cell. The predictions from the four teams look very similar. All provide a relatively good segmentation of the nucleus, with team 3 and 4 overestimating its size slightly more than team 1 and 2. The IoU values, however, are very poor, and for team 3 and 4 are actually not counted as “matches” according to the PQ metric (the matching rule being “$IoU < 0.5$”). Instead, they will be counted as both a “false positive” and a “false negative”, as neither ground truth object nor predicted object will have a corresponding match. In addition, Fig. 10 shows five predicted instances from team 4 that are also not counted as “matches” according to the PQ matching rule. These rejections, however, seem to come mostly from inconsistencies in the annotations themselves. Indeed, for the second and fourth cells, the ground truth annotation appears to cover the entire cell, while the prediction only segments the nucleus, contrary to what is observed for the third and the last cells. On the neutrophil example shown in Fig. 11, we see again nearly identical segmentations with a relatively wide range of IoU values. These kinds of variation observed for negligible differences may mask the impact of “real” errors.

Figure 12 illustrates the problem with the transition between the “panoptic segmentation” task and the “instance segmentation and classification” task. Team 3 is the only one to detect the nucleus of this macrophage but misclassifies it as an epithelial cell. In contrast, none of the other teams detect a nucleus at this location. Team 3’s detection results in both a false positive for the epithelial class, and a false negative for the macrophage class, while the three other teams are only penalized with a macrophage false negative. In a real PS problem, this could not happen because there is no “background” class and any region in the image belongs to a class of interest (see Fig. 2). Therefore, any false negative is always a false positive of another class.

Finally, the lymphocyte example in Fig. 13 illustrates the problem of IoU insensitivity to shape mismatches. Team 1 and 2 have worse segmentations than team 3 and 4 from a biological point of view, because irregularity in segmented shapes could indicate nuclear atypia that is not present. Because the irregularity is very localised and occupies a very small area, it does not penalise IoU, and both teams actually score a bit better than team 4, which overestimates the size of the nucleus by a few pixels but keeps the shape intact.

## Conclusions

To summarise, we have established the following problems with the Panoptic Quality metric for cell nuclei instance segmentation and classification:

1. Because Instance Segmentation and Classification involve a background class for which PQ is not computed, using the per-class F1-score (RQ) as a detection metric is incorrect and results in a more severe penalty for good but misclassified detections than for missed detections.
2. Since nuclei, even at large levels of magnification, are very small objects, the Intersection over Union is a very sensitive metric to use for segmentation and leads to very poor scores for segmentations which are clearly well within the expected variability encountered in an expert annotator.
3. Since IoU is used with a strict threshold of 0.5 for the matching rule and as a consequence of b), many correct detections are missed, resulting in an artificially decreased detection score.
4. Since PQ simply multiplies RQ and SQ, small variations in segmentations, which lead to large changes in SQ, have a similar weight on the overall score as missed detections or misclassifications. Ranking algorithms based on this metric can therefore lead to results that are hard to interpret and may not relate to pathology needs.

It is understandable that researchers seek “catch-all” metrics that allow simple ranking of algorithms for complex tasks. However, these metrics are difficult to interpret, and thus it is difficult to trust the rankings they produce. It should be clear by now that Panoptic Quality is ill-adapted to the particular characteristics of cell nucleus segmentation and classification. It would be more advisable to rank detection, classification, and more adapted segmentation metrics separately (e.g., detection F1-score on a single “nucleus vs background” class, balanced accuracy or AUROC on the classes of the detected nuclei, HD for segmentation). Then, if a single final ranking is needed, a method like the sum of ranks used in the GlaS 2015 challenge^16^ can be used. We previously demonstrated on the MoNuSAC challenge how such a separation could lead to more informative results^11^.

For correct match detection, matching rules based on minimum HD or minimum centroid distance are less likely to lead to false mismatches. While the 0.5 IoU threshold rule has the advantage of directly providing a unique match (i.e. ensuring that it is impossible for a predicted object to be matched with two different ground truth objects, and vice versa), this property can easily be added to other heuristics. For instance, with the minimal centroid distance, the distances between all candidate match pairs can first be computed (within a certain tolerance radius), then sorted so that the matches are assigned in order of their closeness, and any other candidate match from either the ground truth or the predicted object are removed from the candidates list.

We would like to strongly advise challenge organisers and anyone working on nuclei segmentation and classification, to avoid using PQ in the future, and to ensure that their choice of metric avoid the many pitfalls that make it so difficult to trust quantitative results^14^. While the limitations of IoU mostly impact segmentation tasks that target small objects such as cell nuclei, the problem of translation between panoptic segmentation and instance segmentation and classification will impact any task that includes a “background” or “others” class. In such cases, mixing “instance detection” and “instance classification” metrics may be problematic. If the target classes can be grouped into a superclass (such as, for instance, “cell nuclei” or “glands”), the task can be split into “detection of the superclass” and “classification within the detected instances”. Otherwise, it would generally be more appropriate to analyse per-class results separately.

## Data Availability

Accession codes: The MoNuSAC images, annotations, and predictions from the top teams are available from the challenge website (https://monusac-2020.grand-challenge.org/). The NuCLS dataset and annotations are available from the NuCLS website (https://sites.google.com/view/nucls/home). The code used to perform the experiments is available on GitHub (https://github.com/adfoucart/panoptic-quality-suppl).
